# Failure Analysis and Prevention of Extraction Column for Methyl Methacrylate Production: Application of the ‘Safety Design’ Concept

**DOI:** 10.3390/ma14154234

**Published:** 2021-07-29

**Authors:** Sheng-Hui Wang, Yi Gong, Qi Tong, Xiao-Lei Yang, Jia-Hao Shen, Zhen-Guo Yang

**Affiliations:** 1 Department of Materials Science, Fudan University, Shanghai 200433, China; wangwish6@163.com (S.-H.W.); gongyi@fudan.edu.cn (Y.G.); 18110300022@fudan.edu.cn (X.-L.Y.); 2 Shanghai Institute of Special Equipment Inspection and Technical Research, Shanghai 200062, China; 3 Department of Aeronautics and Astronautics, Fudan University, Shanghai 200433, China; tongqi@fudan.edu.cn (Q.T.); 18210290006@fudan.edu.cn (J.-H.S.)

**Keywords:** safety design, failure analysis, extraction column, methyl methacrylate, finite element analysis

## Abstract

To ensure safety and prevent failure of engineering equipment throughout its lifespan, the concept of ‘Safety Design’ is proposed, which covers all the cradle-to-grave phases of engineering equipment, considers at least ten essential factors of failure causes, and conducts root cause analysis at three different scales, in order to proactively control the safety risks before the occurrence of failure rather than passively conduct the remedial measures after failure. Herein, in order to demonstrate how to implement this effective and efficient concept in engineering practice, a case study of failure analysis and prevention is addressed on the extraction column in the production line for methyl methacrylate. Based on the analysis results, the causes were finally determined to be all derived from the stages before operation, including inappropriate design, limited quality inspection of fabrication and installation. Pertinent countermeasures were then proposed from the ‘Safety Design’ point of view, which would not only solve the failure problem for this sole equipment but also contribute to safety risk control of other engineering equipment before operation.

## 1. Introduction

To ensure the safety of engineering equipment, the philosophy of full life cycle (FLC) or the methodology of life cycle management (LCM) is universally accepted and adopted in industry [1,2,3], which covers all the cradle-to-grave phases of equipment, including design, manufacturing, inspection, storage, transportation, installation, commissioning, operation, maintenance, overhaul, shutdown, and decommissioning [4], and manages both the physical factors (degradation, malfunction and failure) and the non-physical factors (asset, economy and staff). Thereinto, design is the foremost phase, which mainly involves siting, configuration, layout, materials, loads and environments, etc., and accounts for 79% of the failure causes of engineering equipment accidents [5]. Operation is the pivotal phase where engineering equipment fulfills its functionalities as designed and is intimately related to materials performance, management strategy and personnel skills. Nevertheless, during the operation phase, engineering equipment is usually obliged to be shutdown temporarily or even long-term if unexpected and/or premature failures occur due to the improper actions taken before this phase, e.g., stress corrosion cracking and crevice corrosion from improper design [6], fracture from improper manufacturing [7], localized erosion from improper installation [8], and atmospheric corrosion from improper storage [9], etc., based on our erstwhile experience.

**Figure 1:**
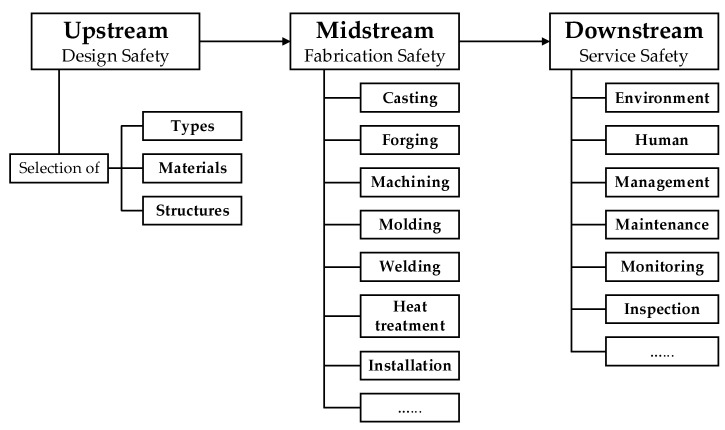
The ‘Safety Design’ concept in the three stages of engineering equipment.

**Figure 2:**
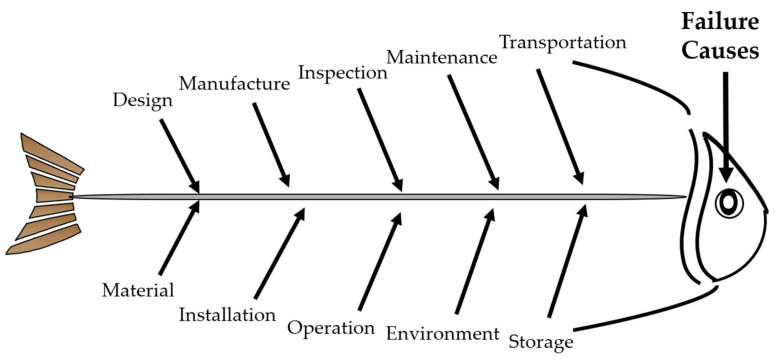
The fishbone diagram of failure analysis for engineering equipment.

Failure analysis is the action commonly undertaken to identify the causes of failed engineering equipment and propose the countermeasures for prevention of the same or similar cases from reoccurrence [10,11,12]. Given that failure greatly influences the reliability, safety, economy and longevity of engineering equipment, it is optimal to proactively control and even eliminate the safety risks before failure [13], rather than to passively implement the remedial measures after failure. This concept can be regarded as the underlying objective of failure analysis and well conforms to the strategy of the FLC philosophy and the LCM methodology in nature. On this basis, we put forward the ‘Safety Design’ concept in brief, which integrates failure analysis and prevention into three aspects. (1) Stage. For convenience, we classify all the cradle-to-grave phases of engineering equipment into three basic stages, including the upstream stage, the midstream stage and the downstream stage, which respectively concentrates on design safety, fabrication safety and service safety, as presented in Figure 1. (2) Scope. To correctly identify the causes of failed engineering equipment, we summarize at least ten essential factors that are necessitated to be taken into account in the failure analysis process, including design, material, manufacture, installation, inspection, operation, maintenance, environment, transportation and storage, and thereby establish the fishbone (Ishikawa) diagram, as displayed in Figure 2. (3) Scale. For the sake of effectiveness, efficiency and economy, in the failure analysis process, we conduct observation of morphologies of failed engineering equipment at three different scales in sequence, including a preliminary analysis in the macroscopic scale (>10^−2^ m) to determine the failure modes (e.g., corrosion, wear, fracture and distortion), a detailed analysis in the mesoscopic scale (10^−5^~10^−2^ m) to confirm the failure forms (e.g., pits, dents, cracks, striations), and an in-depth analysis in the microscopic scale (<10^−5^ m) [14] to differentiate the failure mechanisms (e.g., pitting corrosion, abrasive wear, stress corrosion cracking, fatigue). Once the failure causes are identified at the upper scales, no further analysis is necessary at the subsequent lower scales.

To demonstrate how to put this ‘Safety Design’ concept into practice for engineering equipment, this paper presents a case study of failure analysis and prevention on one piece of petrochemical equipment, the extraction column in the production system of methyl methacrylate (MMA). According to the design requirement, the projected lifetime of it was at least twenty years. However, unexpected abnormal wear was discovered on the inner wall of its shell during the routine overhaul after serving for only four years. Since the normal operation of the extraction column had been affected, which would probably even threaten the safety of the whole system, it was therefore shut down at once and the standby one was put into service. At the same time, in order to prevent the reoccurrence of the same case, failure analysis was urgently required as well. By referring to our previous experiences on cause analysis of failed engineering equipment in the last several years, comprehensive investigation and systematic analysis was immediately carried out. As a result, in addition to the cause identification, the countermeasures focusing on the phases before operation were also proposed for failure prevention, including design modification, manufacturing improvement and installation optimization. This paper is a sequel to our previous cause analysis of localized abnormal wear for this same piece of equipment [15], but it can be taken as an example to demonstrate the ‘Safety Design’ concept in engineering practice. The achievements of this paper would not only enrich the database of failure analysis cases [16,17,18] but also have reference value for scholars in relevant fields to utilize this ‘Safety Design’ concept in the failure analysis and prevention of engineering equipment.

## 2. Experimental

**Figure 3:**
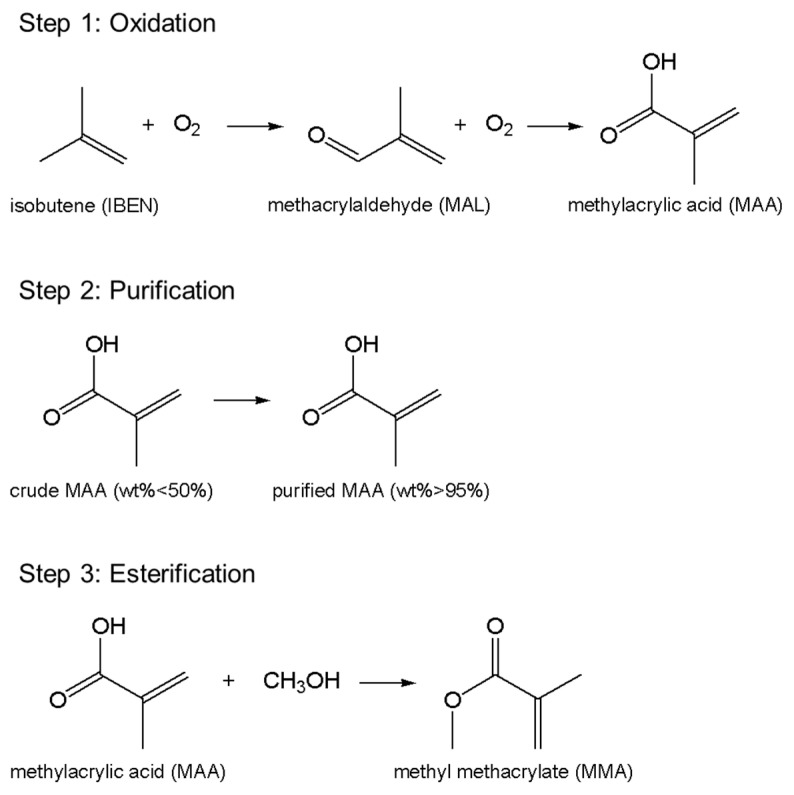
The main steps of C4 direct oxidation method to produce MMA.

**Figure 4:**
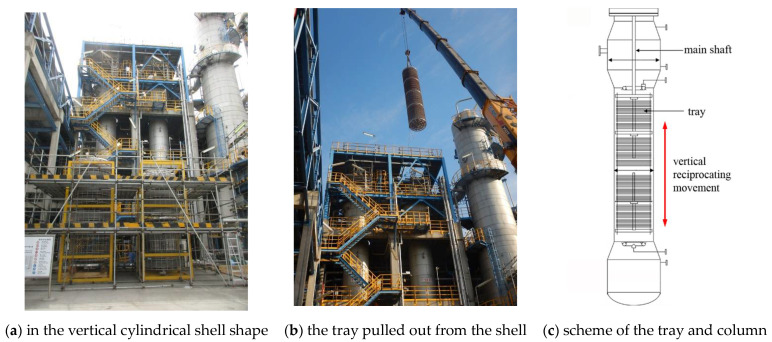
External appearances of the extraction column.

**Figure 5:**
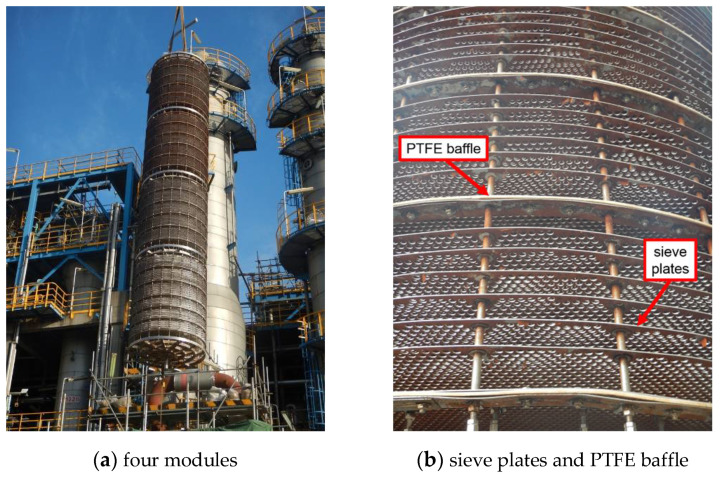
External appearances of the tray.

Herein, one certain petrochemical company adopts the C4 direct oxidation method to produce MMA with annual output of over 100,000 tons. This method consists of three main steps in sequence, as listed in Figure 3. It initiates from the oxidation of the C4 feedstock isobutene (C_4_H_8_), as the name implies; progresses with the purification of the intermediate product methylacrylic acid (MAA); and terminates at the esterification of MAA for producing the end product MMA. The extraction column is located in the second step, and its main functionality is to purify the crude MAA by mixing the MAA aqueous solution with the extraction solvent heptane (C_8_H_18_). As exhibited in Figure 4a, it is in the shape of a vertical cylindrical shell with the size of 21,300 × 2000 × 10 mm^3^ (height × diameter × thickness), and the matrix material is 316 stainless steel. Inside it, an active tray is installed, as shown in Figure 4b,c, which is controlled by the main shaft in the center of the extraction column to achieve a purification function through vertical reciprocating movement with the liquids under the designed frequency of 90 per minute. Furthermore, it is displayed in Figure 5a that the tray is further divided into four modules along the vertical direction. Each module contains 36 sieve plates, and the plates are separated into groups of 6 by a baffle made of polytetrafluoroethylene (PTFE) for protection of the tray from direct collision onto the shell, as shown in Figure 5b. According to the design drawing, the diameter of the PTFE baffle is 1985 mm, that is, a 7.5 mm-wide clearance, (2000−1985)/2, is reserved between the PTFE baffles and the inner wall of the shell.

**Figure 6:**
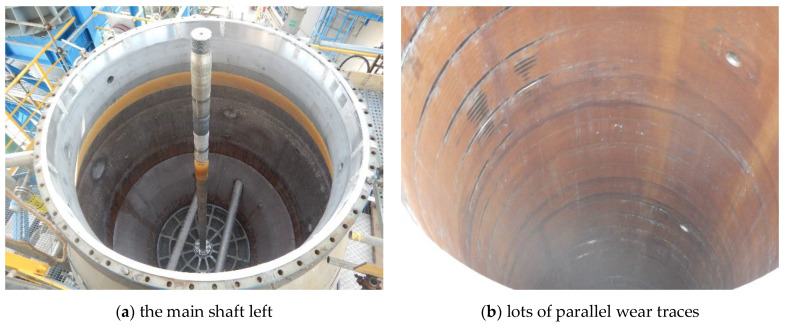
External appearances of the shell inner wall after pulling out the tray.

After serving for four years, it was detected that the operational frequency of the tray gradually decreased. As a matter of fact, the phenomenon of frequency decrease had already been anticipated in the design because the products and byproducts would inevitably deposit and foul on the sieve plates of the column, and consequently, the actual operational frequency of the tray was set and maintained in the range of 60 to 70 per minute. However, this time the frequency decreased by half, falling into the range of 30 to 40 per minute, and could not be increased any more. Undoubtedly, the extraction efficiency of the column and thereby the production capacity of the system would be affected if this problem was not adequately solved. Hence, the extraction column was shut down at once, and the standby one was put into service. In addition, an inspection was conducted by the field personnel on the interior of the failed column to identify the causes. It was reported by them that some of the PTFE baffles, even the sieve plates, were directly contacting with the shell of the column, and lots of strip-shaped traces were left on the inner wall of the shell. It is explicit that the direct contact between the baffles/sieve plates and the shell must be the reason for the frequency decrease of the tray, and the problem was thus accordingly shifted to identify the causes and consequences. To this end, the whole tray was pulled out from the shell for further investigation (Figure 6a). Thereby, lots of parallel wear traces indeed emerged on the inner wall of the shell, as displayed in Figure 6b.

In normal conditions, it is impossible for the tray to contact the inner wall of the shell on account of the 7.5 mm-wide clearance. Hence, the first idea of the field personnel was to ascribe the wear to the inclination of the shell. If so, the whole extraction column must be completely refurbished or even replaced and would induce unaffordable economic losses to the company. To ascertain/eliminate that cause, failure analysis was then carried out by us.

Since this extraction column was an intact pressure vessel, it was not feasible to cut material samples from any component of it, especially the shell. Consequently, according to the ‘Safety Design’ concept, investigation could only be carried out at the macroscopic scale. Based on the analysis results, the safety risks in the downstream service stage were excluded. In other words, the failure causes were primarily derived from the other two stages, i.e., the upstream design stage and the midstream fabrication stage. Thus, to demonstrate the way to practice the ‘Safety Design’ concept, the following paragraphs will address the failure causes identification of this extraction column and propose countermeasures for failure prevention of similar engineering equipment in the future.

## 3. Results and Discussion

### 3.1. Failure Analysis

**Figure 7:**
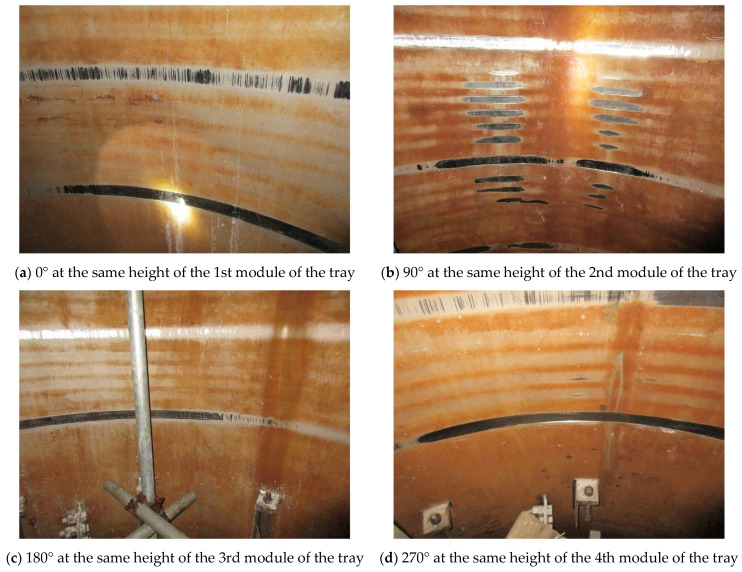
Wear traces on the inner wall of the shell.

The first step was to investigate the possibility of inclination of the shell of the extraction column. Thus, visual inspection was conducted on the inner wall of the shell. As displayed in Figure 7, the wear traces were discovered distributing on all the four main directions of 0°, 90°, 180° and 270° along the circumference, and on all the heights equal to the four modules of the tray. That is, the degree of wear between the different directions and heights showed no obvious difference; only in some areas, the trace depth reached as deep as 2.5 mm (Figure 7b). This phenomenon was solid evidence of excluding the possibility of inclination of the column shell, because it would have caused wear traces in one direction, and the wear extent would have gradually decreased downwards along the height.

**Figure 8:**
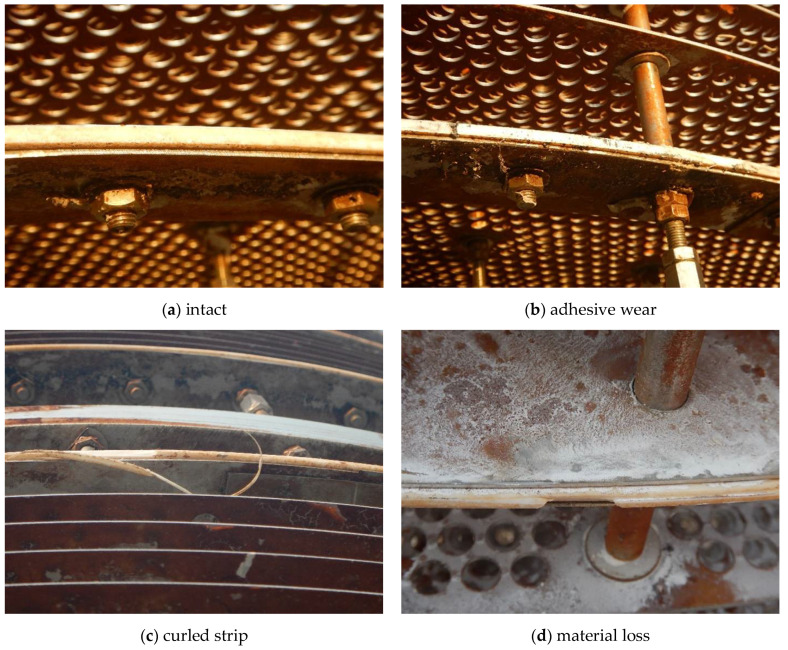
Diverse wear morphologies on the PTFE baffles.

While for the tray, wear phenomenon was also discovered. Compared with the intact PTFE baffles that had not suffered wear (Figure 8a), the ones that had suffered wear exhibited diverse morphologies such as adhesive wear (Figure 8b), curled strip (Figure 8c) and material loss (Figure 8d), etc., indicating quite severe wear.

Thus far, it can be basically ascertained that severe wear had indeed occurred between the tray (i.e., the sieve plates and the PTFE baffles) and the inner wall of the column shell, but the possibility of inclination of the shell was excluded. In other words, the causes should be revealed from other aspects.

### 3.2. Design Safety Analysis

As mentioned above, on the main shaft (as shown in Figure 6a) there is a tray equipped for extraction, which contains a total of 5 PTFE baffles and 144 sieve plates. Per the design drawing, the diameters of the baffles and the plates are 1985 and 1920 mm, respectively, while that of the main shaft is only 88.9 mm, less than 5% of the former two. Then, a problem naturally emerges of whether the main shaft has sufficient structural stiffness to hold the tray under the operational conditions. Hence, a finite element analysis was performed.

**Figure 9:**
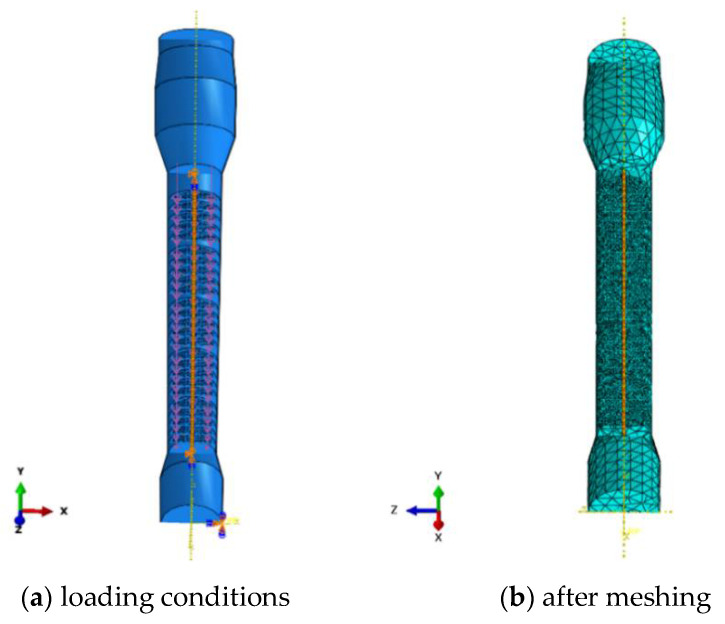
The finite element model of the extraction column.

Since the weight of the tray has little effect on the main shaft, the load taken into consideration was mainly the impact from the two fluids, i.e., the MAA aqueous solution flowing downwards and the extraction solvent heptane flowing upwards. By using ABAQUS, the numerical model was established according to the design drawing, and only the main shaft with the tray involved in the vertical reciprocating movement was taken into consideration, as presented in Figure 9a. The impact of fluids was time-averaged and applied as static pressure on the surfaces of the sieve plates and the PTFE baffles, which can be deduced under the principle of mass and momentum conservation, as seen in Equation (1).

(1) $$P=\frac{1}{2A_{f}}\left(1-A_{f}^{2}\right)\left(2g\Delta h+A_{f}^{2}v^{2}\right)$$

where *P* is the momentum (kg·m/s), *A_f_* is the area ratio (dimensionless), *g* is the gravitational acceleration (m/s^2^), Δ*h* is the height difference (m) and *v* is the velocity (m/s). All the components were made of 316 stainless steel except the PTFE gasket, and the materials parameters (Table 1) could be obtained from the materials handbook.

In the model, the shell was considered rigid and coarsely meshed with 7223 three-dimensional rigid triangular elements (R3D3 from Abaqus element library). To prevent the sieve plates and the PTFE baffles from yielding under the static pressure, they were modeled by stiff materials compared with the main shaft, but all these three components were finely meshed with 242,130 tetrahedral elements C3D10, seen in Figure 9b. It should be noted that it generally will save much computational cost to use structural elements instead of continuum elements. Nonetheless, the coupling of degrees of freedom between nodes of different types of structural elements is very complicated and may lead to unrealistic results. In this study, we consider the contact of the plates and baffles with the rigid shell and the deformable shaft. It makes the construction of the corresponding constraint equations of the nodal degrees of freedom quite complex under the Kirchhoff assumption of shells. This is the reason for choosing continuum elements C3D10 for the simulation. As for the boundary conditions, the main shaft was pinned at both the upper and the lower ends of the shell, and in the analysis, the periphery of the tray and the inner wall of the shell were treated as contact pairs.

**Figure 10:**
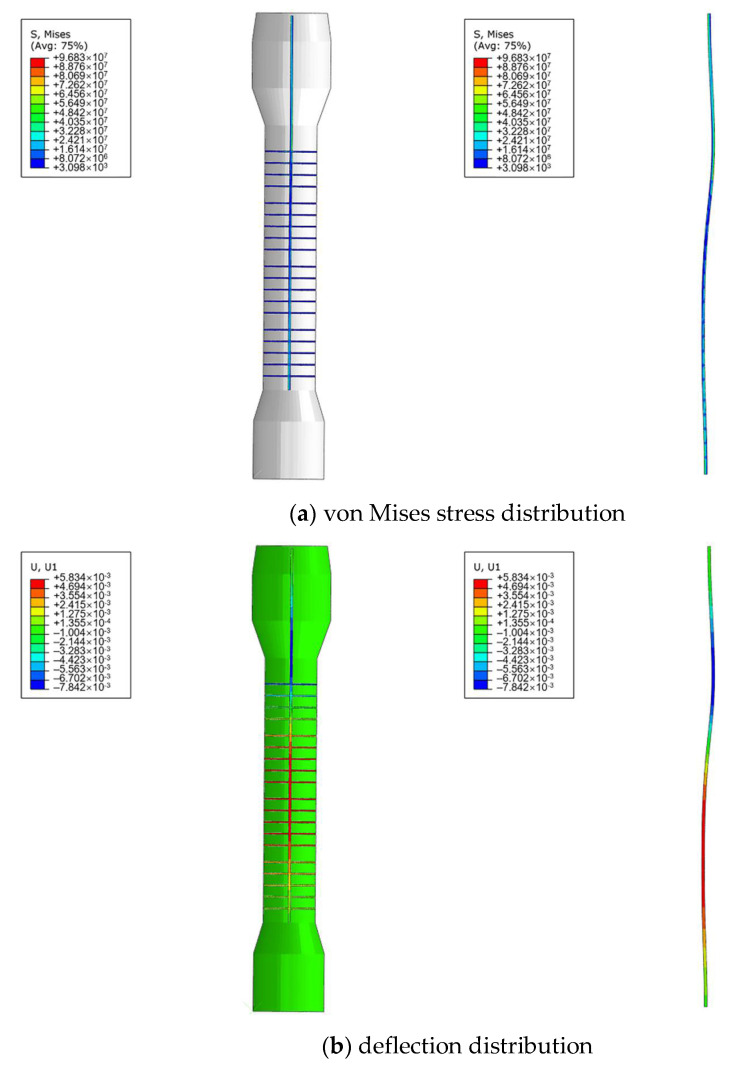
Finite element analysis results of the main shaft.

Figure 10a presents the von Mises stress distribution of the main shaft. It shows that the largest stress located at the connection between the shaft and the first module of the tray, which should be attributed to the higher pressure upon the tray from the top and the boundary condition at the upper end. It also reveals that the stress distribution in the middle of the shaft was homogeneous and the stress in the major bulk of it was still under the yield strength. However, as shown in Figure 10b, deflection did occur on the main shaft under the impact of the fluids, and the maximum radial deflection ranged from −5.8 to 7.8 mm. Obviously, it had partly exceeded the clearance (7.5 mm) and, consequently, induced wear on the shell. Furthermore, the result indicated that the maximum deflection was located at the same height with all the four modules of the tray, which accorded with the phenomenon observed in Figure 7 that the wear extent on the shell was of average severity along the vertical direction.

Since structural stiffness is the ratio between load and maximum deflection, as presented in Equation (2), it was determined that the main shaft with the current diameter did not have sufficient stiffness based on the analysis results. Then a new problem emerges of what is the minimum required value? Thus, further investigation was carried out to obtain the relation between the diameter of the main shaft and the largest deflection under the same loading condition.

(2) $$K=\frac{P}{\delta }$$

where *K* is the structural stiffness (N/m), *P* is the load (N), *δ* is the maximum deflection (m).

**Figure 11:**
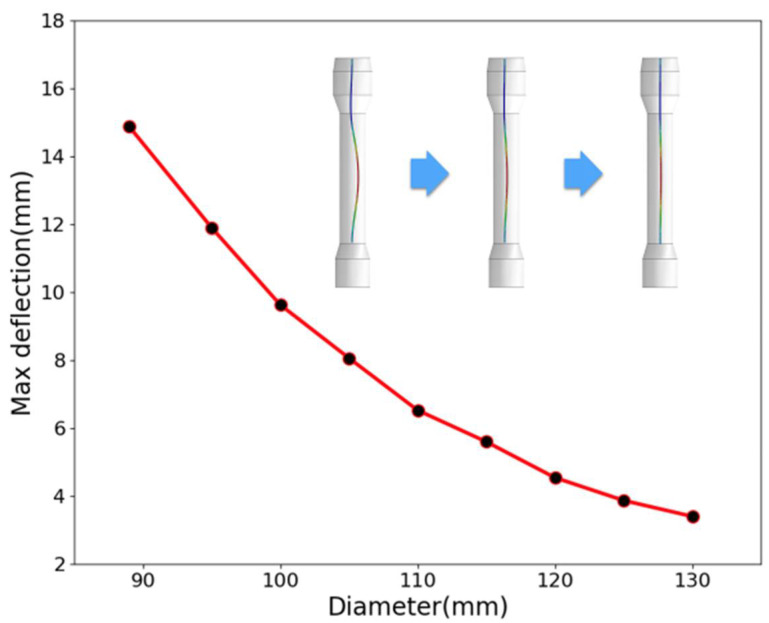
Maximum deflection versus the diameter of the main shaft.

As shown in Figure 11, without the limitation of the shell, the maximum deflection of the main shaft decreases monotonically with its diameter. In other words, the structural stiffness will increase with the diameter of the main shaft. Quantitatively, the maximum deflection can be reduced to 7.5 mm when the diameter reaches 105 mm. That is, if the current operational condition cannot be changed, 105 mm should be the minimum required diameter of the main shaft for prevention of deflection and subsequent wear upon the shell. To sum up, it could be proposed from the finite element analysis results that the inappropriateness of the current design was the root cause for abnormal wear on the shell.

### 3.3. Fabrication Safety Analysis

**Figure 12:**
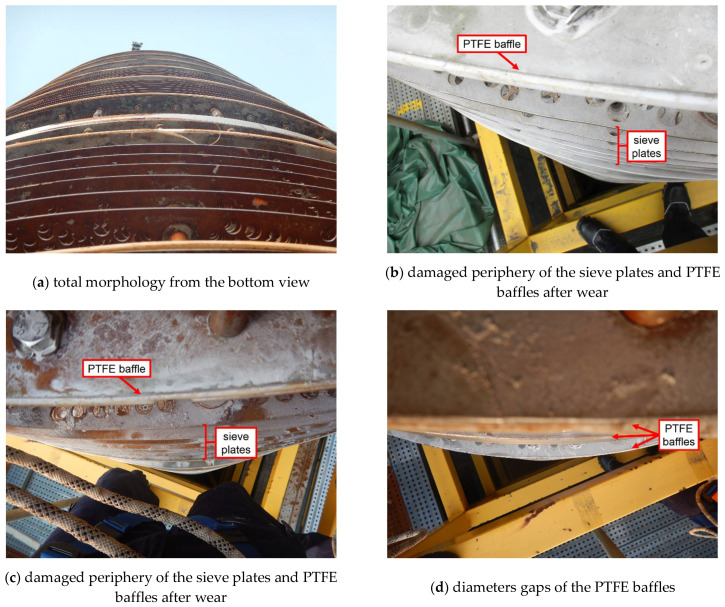
External appearances of the tray.

After analysis of the main shaft, now it comes to the tray. During the visual inspection, it was found that the shape of the sieve plates and the PTFE baffles was actually not perfectly round in general, seen in Figure 12a. That is, there existed some ovality. Furthermore, from the top view of the tray (Figure 12b,c), it was observed that the periphery of the sieve plates was partly damaged after wear; while for the PTFE baffles, the differences between their diameters even reached two to three centimeters, as presented in Figure 12d.

These phenomena clearly pointed one of the causes of wear to limited quality inspection of fabrication of the sieve plates and the PTFE baffles. Although the extraction function of the tray does not depend on the dimensional precision of the baffles and the sieve plates and a 7.5 mm-wide clearance is particularly reserved between them and the shell, the wear extent upon the shell will be aggravated due to the ovality of these two components once wear is initiated from deflection of the main shaft. In brief, although only ovality of the baffles and the sieve plates themselves would not result in wear on the shell of the extraction column, it was indeed an added factor for such severe wear due to limited quality inspection of fabrication.

**Figure 13:**
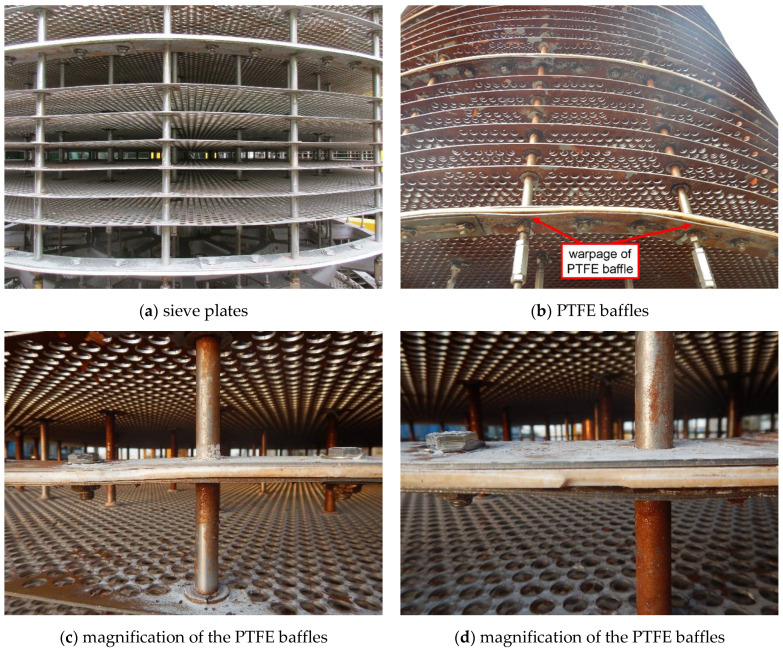
External appearances of warpage on the sieve plates and the PTFE baffles.

In addition to ovality, another defect we discovered on the sieve plates and the PTFE baffles was warpage, as seen in Figure 13a,b. Especially for the baffles, part of the plate stacks and the PTFE gasket had already separated (Figure 13c), and the gasket had also suffered severe wear (Figure 13d).

It is not difficult to imagine that the warpage of such two components will narrow their clearance between the shell and, consequently, further aggravate the wear extent upon the shell. With regards to the causes of warpage, it was inferred that during the process of installation, the bolts for fixing the sieve plates and the PTFE baffles were assembled forcibly, leaving excessive tightening force on the two components. Thus, it was determined that limited quality inspection of installation was also an added cause for severe wear on the shell.

### 3.4. Effectiveness Verification

Thus far, it is clear that insufficient structural stiffness of the main shaft due to inappropriate design was the root cause of deflection of the main shaft, which subsequently induced abnormal wear on the inner wall of the extraction column shell. In addition, ovality and warpage from limited quality inspection of fabrication and installation of the sieve plates and the PTFE baffles further aggravated the extent of wear. With regards to the types of wear, adhesive wear and fatigue wear should be mainly ascribed to, whose detailed mechanisms could be referred to as classic monographs [19] and would not be repeatedly discussed in this paper. However, coincidently, it can be summarized that all the three causes occurred in the phases prior to operation of the extraction column, i.e., design, manufacturing and installation. By the way, this wear phenomenon was also discovered in the other standby extraction column in the next routine overhaul, but the extent of it was not as severe because it had only served for one operation cycle.

**Figure 14:**
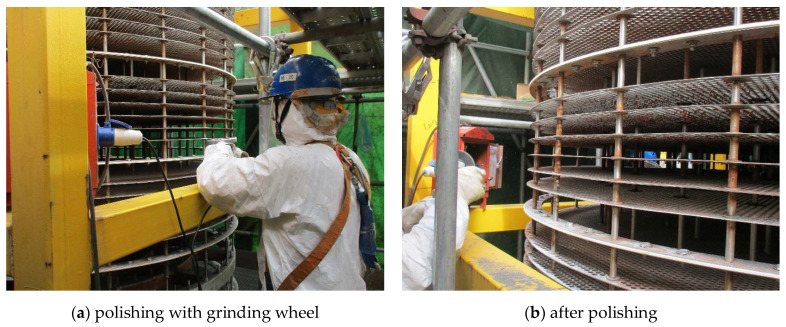
Field personnel polishing the periphery of the sieve plates and the PTFE baffles.

Next is addressing the countermeasures. As for insufficient structural stiffness of the main shaft, on the basis of the finite element analysis results, to replace it with a new one with a diameter larger than 105 mm will certainly take effect. However, it will undoubtedly be timely and expensive, which is not acceptable for the company. Thus, the focus was then shifted to the measures that would not change the current service conditions of the extraction column and the current dimension of the main shaft. In other words, the measures should take into full consideration the fact that deflection of the main shaft would be inevitable. Then, we considered taking action from the other side. As indicated in Figure 11, the maximum deflection of the main shaft with a current diameter of 88.9 mm is about 15 mm, twice the clearance (7.5 mm) between the PTFE baffles and the shell of the column. Therefore, why not enlarge the clearance to tolerate the deflection? Thus, we suggested reducing the diameters of the sieve plates and the PTFE baffles by 5 to 10 mm. This time, it was accepted and immediately implemented by the company, as shown in Figure 14. From then on (over three years have passed), the problem of abnormal wear was no longer reported.

In terms of the other two causes, including ovality and warpage of the sieve plates and the PTFE baffles due to limited quality inspection of fabrication and installation, the suggestions were relatively simple and directly accepted by the company, i.e., improving the quality of manufacturing and installation to eliminate ovality and warpage, respectively. Of course, they would only be beneficial to the extraction columns built in the future.

## 4. Conclusions and Countermeasures

Failure analysis was carried out for identification of the causes of abnormal wear on the inner wall of the extraction column shell, and the results pointed to the three phases prior to operation, which vividly demonstrated the ‘Safety Design’ concept for engineering equipment:

(1) In the design phase, the diameter of the main shaft was not large enough to provide necessary structural stiffness, so wear was induced due to deflection of the shaft.
(2) In the fabrication phase, the quality of the sieve plates and the PTFE baffles was not sufficiently inspected; therefore, ovality was left and aggravated the wear extent on the shell.
(3) In the installation phase, the warpage was formed on the sieve plates and the PTFE baffles because of limited quality inspection, which also aggravated the extent of wear.

Based on the above conclusions, countermeasures were put forward point by point for failure prevention of this extraction column:

(1) The periphery of the sieve plates and the PTFE baffles should be polished to reduce their diameters for tolerance of the main shaft deflection.
(2) The quality of manufacturing and installation of the sieve plates and the PTFE baffles must be improved for the elimination of ovality and warpage.
(3) Although no faulty operation from the field staff was found this time, training and management still need to be further strengthened, e.g., when abnormal signals are detected during the operation phase in the future, the equipment should be immediately stopped for root causes analysis.

## Figures and Tables

**Table 1:** Physical and mechanical properties of 316 stainless steel.

Properties	Density (kg/m^3^)	Elastic Modulus (GPa)	Tensile Strength (MPa)	Yield Strength (MPa)	Elongation (%)
Values	7980	~190	~580	~205	≥40

## Data Availability

Not applicable.
